# Diagnostic utility of ultrasonography for thoracic and abdominal bacterial and parasitic diseases in ruminants: a comprehensive overview

**DOI:** 10.3389/fvets.2024.1435395

**Published:** 2024-09-02

**Authors:** Mohamed Tharwat, Takeshi Tsuka

**Affiliations:** ^1^Department of Clinical Sciences, College of Veterinary Medicine, Qassim University, Buraidah, Saudi Arabia; ^2^Department of Veterinary Clinical Medicine, Joint Department of Veterinary Medicine, Faculty of Agriculture, Tottori University, Tottori, Japan

**Keywords:** bacteria, infectious disease, parasitism, ruminant, ultrasonography

## Abstract

This review article describes the roles of ultrasound in assessing thoracic and abdominal infectious diseases, mainly bacterial and parasitic ones that affect farm animals, including cattle, camels, sheep, and goats. Ultrasonography is a non-invasive imaging technique used to diagnose infectious diseases affecting the cardiovascular, respiratory, digestive, urinary, and hepatobiliary systems. In cases of thoracic and abdominal infections, ultrasound typically reveals abnormalities in echogenicity and echotexture, the presence of unusual artifacts, and mass formation exerting pressure on surrounding structures. Inflammatory and degenerative changes within the viscera can be identified ultrasonographically by comparing the echogenicity of affected areas with that of the surrounding normal parenchyma, such as in fascioliasis. Bacterial and parasitic infections often result in capsular mass lesions with anechoic contents, as observed in hydatid cysts and cysticercosis, or varying echogenic contents, as observed in liver abscesses. Effusions within the pericardium, pleura, and peritoneum are common ultrasonographic findings in infectious thoracic and abdominal diseases. However, these effusions' echogenicity does not always allow for clear differentiation between transudates and exudates. The routine use of ultrasonography in the evaluation of the chest and abdomen in affected or suspected ruminants is highly beneficial for detection, guiding therapeutic decisions, assessing prognosis, and aiding in the eradication of highly contagious diseases that cause significant economic losses.

## 1 Introduction

For over 50 years, physicians have been using ultrasonography (US) to aid in diagnosis and guide procedures in day-to-day examinations. US is a simple, safe, and non-invasive tool implemented broadly in general practice (1). There is no doubt that early diagnosis of diseases in animals will affect treatment progress. Therefore, any delay in diagnosis can lead to unwanted complications. From this standpoint, developing new methods to diagnose diseases in their early stages is necessary. Diagnostic imaging modalities, including radiography, computed tomography (CT), and US, are used in veterinary medicine for both diagnosis and therapeutic purposes. However, all modalities except the US require special precautions, and CT is performed only in fixed sites and more developed countries (2).

In contrast, the US is routinely used in veterinary medicine to investigate various bovine disorders (3–6). The US supplements clinical and laboratory examinations by providing additional information on thoracic and abdominal disorders and verifying antemortem conditions. US is well-tolerated in unsedated animals, allowing serial examinations to monitor disease progression and treatment response or practice scanning techniques. This article reviews the effectiveness of US in assessing infectious diseases, mainly bacterial and parasitic, that affect various ruminants, including cattle, buffaloes, camels, sheep, and goats.

### 1.1 Scanning technique of thoracic and abdominal US

The required US frequency in the transducer used for both sides of the chest is ≤ 5.0 MHz for adult cattle and buffaloes (7–11) and ≥5.0 MHz for younger calves and small ruminants (2, 12–15), ensuring good quality thoracic US.

US images of the lungs and pleura can be acquired by scanning the intercostal spaces between the 5th and 12th ribs for cattle and between the 3rd and 11th ribs for buffaloes (11, 16). For younger calves, intrathoracic structures on both sides can be scanned between the 3rd and 10th ribs on the left and between the 1st and 10th ribs on the right (14). The anatomical locations to scan the heart include the intercostal spaces between the 3rd and 6th ribs in buffaloes (11), a wider range than the 3rd to 4th intercostal spaces used in cattle (7).

When scanning the abdomen of adult cattle and buffaloes, a lower US frequency between 3.5 and 5.0 MHz is required (8, 9, 11, 17–20). High-quality images can be obtained using a ≥5.0 MHz transducer for the abdominal US in younger calves and small ruminants (19, 21, 22).

The liver can be imaged by US scanning at the right-sided chest region between the 9th and 11th ribs in cattle (18, 23), between the 9th and 12th ribs in buffaloes (11), and between the 7th and 13th ribs in goats and sheep (24–26). In goats and sheep, the liver is observed adjacent to the echogenic line of the diaphragm at the level of the 10th rib (24, 25). The gall bladder is visualized within the liver parenchyma in the US, taken at the 11th to 12th intercostal spaces in buffaloes and at the 9th to 11th intercostal spaces in goats and sheep (11, 24, 25). The common anatomical location to scan the reticulum is the ventral abdominal wall, just caudal to the xiphoid cartilage in cattle and small ruminants (24). In buffaloes, the reticulum can also be examined by US scanning at the ventral thorax aspect on both sides of the sternum within the 6th to 8th intercostal spaces (11). The scanning position for the abomasum in cattle is the ventral midline and paramedian regions of the abdomen at ~10 cm caudal to the xiphoid process (17). The small intestine can be detected on the abdominal US when scanned on the right side between the 8th rib and pelvis and from the areas of the transverse processes of the vertebrae to the linea alba (17). The intercostal spaces between the 10th and 12th ribs and between the 9th and 12th ribs are the scanning locations allowing visualization of the descending duodenum and the jejunum and ileum in cattle, respectively (17). In adult cattle and buffaloes, transrectal scanning is necessary for US observations of the left kidney and urinary bladder, whereas percutaneous US scanning allows visualization of the right kidney (11, 19, 27–30). Percutaneous US scanning can examine the left kidney at the caudal left paralumbar fossa in some camels, younger calves, and lean adult cattle (28, 30–32). Additionally, percutaneous US can demonstrate the urinary bladder when applying the transducer to the ventral aspect or both flanks of the caudal abdomen in younger calves or small ruminants (33, 34).

## 2 Chest

### 2.1 Pneumonia/pleuropneumonia

Inflammation of the pulmonary parenchyma primarily affects the alveoli and is usually accompanied by inflammation of the bronchioles and often by pleuritis. Clinically, the condition is characterized by cough, abnormal respiratory sounds, polypnea, and changes in the depth and type of respiration (35). It may be caused by viruses, such as respiratory syncytial virus, retroviruses, paramyxovirus, oncogenic beta-retrovirus, capripox virus, and a small ruminant lentivirus group (known as a Maedi-visna); bacteria, such as *Pasteurella multocida* and *Mannheimia haemolytica*; fungi, such as *Cryptococcus neoformans*; parasites, such as *Dictyocaulus filaria, Protostrongylid rufescens*, and *Echinococcus granulosus*; as well as mycoplasma, chlamydia, and rickettsia (35, 36). Infection with *Fusobacterium necrophorum* typically induces chronic suppurative pneumonia, often via various infection entry points such as inhalation and hematogenous spread in younger animals. This occurs when the animals present with chronic weight loss, depression, tachypnea, cough, and mucopurulent nasal discharge. Pleuritis, or pleurisy, is inflammation of the pleura. It is often associated with pulmonary parenchyma inflammation, known as pleuropneumonia (37). Contagious caprine pleuropneumonia is a severe disease of small ruminants, especially goats, found in African and Asian countries (13, 38, 39). The disease is caused by *Mycoplasma capricolum* subsp. *capripneumoniae* infections (13, 38, 39). The acute and subacute forms of the disease are characterized by unilateral fibrinous pleuropneumonia with severe pleural effusion (13, 39, 40).

Although auscultation is considered an important component of veterinary clinical examination, it cannot fully provide diagnostic evidence of pneumonia or pleuropneumonia regarding the severity, extension, and localization of lung pathology (40, 41). Diagnosis of contagious caprine pleuropneumonia relies on clinical and necropsy observations that should be confirmed by laboratory tests (40). Due to the difficulty of isolating this pathogen, molecular techniques are preferred for laboratory confirmations (13). Therefore, ancillary diagnostic approaches are essential to confirm provisional diagnoses from clinical examinations (8).

**Figure 1:**
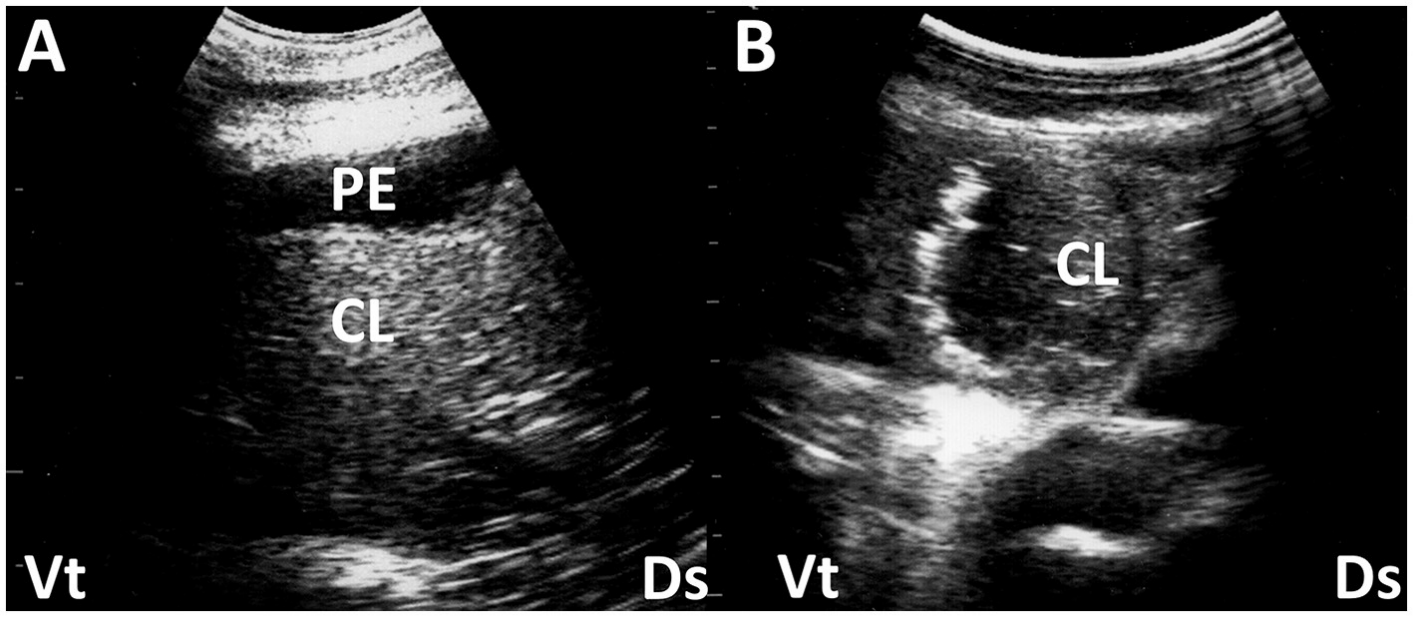
Ultrasonograms **(A, B)** of a consolidated lung (CL) in two camel calves with pneumonia. **(A)** Echogenicity in CL is increased heterogeneously. Anechoic pleural effusion (PE) is also evident. **(B)** In a consolidated lung, small pockets of gas often remain, seen as small hyperechoic areas. Ds, dorsal; Vt, ventral (45).

**Figure 2:**
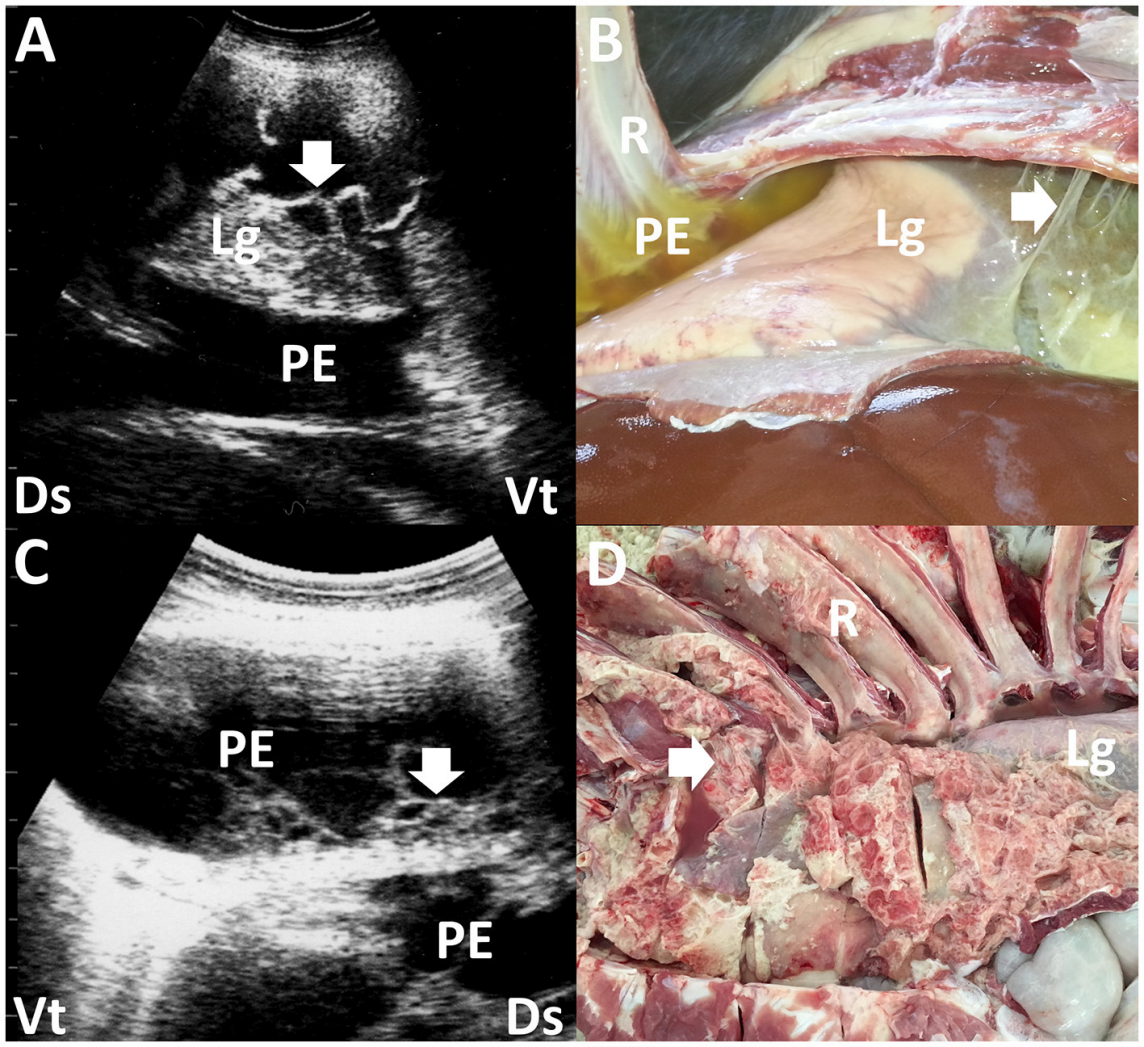
Ultrasonograms and macroscopic views show contagious caprine pleuropneumonia in a caprine case **(A, B)** and fibrinous pleuropneumonia in a camel calf **(C, D)**. **(A)** Echogenic fibrinous strands (arrow) are floating within an anechoic pleural effusion (PE). Compressed lung structures are demonstrated as echogenic. Lg, lung; Ds, dorsal; Vt, ventral. **(B)** Fibrinous adhesion (arrow) between the lung (Lg) and pleura and the accumulation of clear yellow pleural effusion (PE) is evident. R, rib (13). **(C)** Webbed fibrinous strands (arrow) are floating within hypoechoic pleural effusion (PE). Ds, dorsal; Vt, ventral. **(D)** Fibrous adhesive scars (arrow) are evident over the surface of the lung (Lg). R, rib (45).

Thoracic US is very helpful for diagnosing pneumonia due to its ability to distinguish between pathological lung echotextures and the specific US pattern of normal lungs (16, 41–43). Air-filled lung tissues are a strong reflector of US waves, trapping these waves between the lung tissues and the surface of the transducer, generating a reverberation artifact (16, 44). The change in the echotexture of lung structures, resembling that of liver parenchyma, represents pulmonary consolidation (16, 45) (Figure 1). Air-filled small bronchi appear as hyperechoic foci within the hypoechoic echotexture of affected lung structures (16). Detecting consolidation lesions deep within normal air-filled lung structures can be difficult due to reverberation artifacts (16). In cases of severe pneumonia, a US examination reveals hypoechoic zones on the lung surface, representing superficial fluid alveolograms with a comet-tail artifact (45). Accumulating a small amount of effusions within the space between the parietal and visceral pleura can also generate a comet-tail artifact (2, 12, 15). The presence of adhesions between the parietal and visceral pleura can be indicated by the disappearance of the gliding sign, which is the normal sliding movement of the lung within the thoracic cavity during respiration (16). US findings in small ruminants with contagious caprine pleuropneumonia often include the presence of gas echoes within the pleural cavity or abscess formation (13). The mixture of abnormal lung and pleural echotextures is usually detected unilaterally in many caprine cases because the pleural sacs do not communicate; pleural fluid readily transmits sound waves and, therefore, appears anechoic (13) (Figures 2A, B). In cattle, pleuropneumonia is associated with an extension of infectious inflammation over the pleura of the lung, thoracic wall, and diaphragm, mostly affecting one side of the body (37). In camels, US of the thoracic cavity can also detect pleural effusion and aid in determining the prognosis of the diseased animal (45). In severe cases, it shows bilateral heterogeneous pleural effusions with fibrin threads (45). Fibrin appears as filmy and filamentous strands floating in the effusion with loose attachments to the pleural surfaces; pockets of fluid separated by fibrin are commonly imaged (45) (Figures 2C, D). Regarding active phase detection, the use of US appears to be as effective as thoracic radiography (14). The routine use of US may detect the subclinical phase of bovine respiratory disease (43).

### 2.2 Pleural effusion

Pleural effusion is not only a local pleural disease caused by primary pleural infection via local or systemic routes but also a concurrent condition associated with various cardiorespiratory diseases such as pneumonia and pericarditis (45, 46). Pleural effusion is also a non-specific finding associated with heart failure and decreased blood circulation (7). In bovine cases, echogenicity in pleural effusion is hypoechoic, suggesting pleural transudates. An increase in echogenicity suggests empyema, which contains varying amounts of cellular contents, debris, and fibrin (37). The US is useful in guiding thoracocentesis in collecting pleural effusion (37).

### 2.3 Lung cysts

Echinococcosis is one of the most important zoonoses caused by metacestodes of *Echinococcus granulosis* infecting humans and livestock (26, 47, 48). Early detection of this disease is required during the breeding stages. Echinococcosis can induce the formation of single or multiple lung cysts and liver cysts, which are characterized in the US by cavitary masses lined with thin or thick walls containing anechoic to hyperechoic fluids (48). Cystic lesions can be identified within both the lung and liver structures in approximately half of the affected cases (48). Additionally, the US detection sensitivity and specificity for cystic lesions are 88.7 and 75.9%, respectively (47). The severity of respiratory disturbance appears to correlate with the size and multifocal nature of the cystic lesions (48). The US is useful in diagnosing echinococcosis by guiding fine needle aspiration of fluids within the cystic mass, which allows for macroscopic detection of protoscoleces in the germinal cyst layer (48).

### 2.4 Lung and pleural abscessation

**Figure 3:**
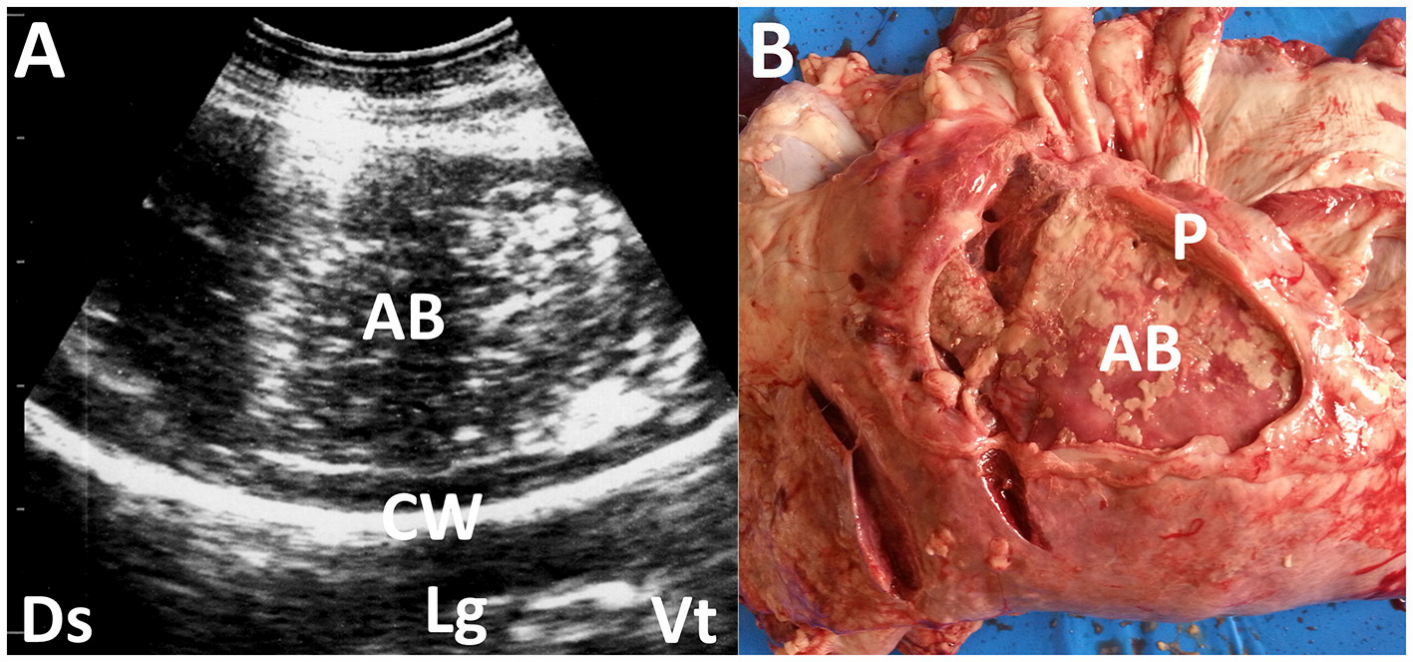
Ultrasonogram **(A)** and macroscopic view **(B)** showing pleural abscessation (AB) in a buck. **(A)** The capsular mass with a size of >3 cm includes the heterogeneous, hyperechogenic contents within the hyperechoic capsular wall (CW). Lg, lung; Ds, dorsal; Vt, ventral. **(B)** The AB mass is derived from the thickened pleural walls (P).

Lung abscesses commonly occur in association with the chronic phase of various types of pneumonia. Pleural abscesses can occur due to primary pleural infections from various causes, including trauma or secondary foci extended from lung infections. An abscess can be demonstrated as a well-defined cavitary mass enveloping its contents with variable echogenicity if it develops within the affected lung and pleura (16). The cavitary mass can generate acoustic enhancement (45). The formation of small pulmonary nodules is one common US sign of bronchopneumonia, representing small abscesses and inflamed or necrotic foci (16). Pleural abscesses can cause compression of the peripheral lung structures, depending on their sizes (45) (Figure 3). The echotexture of the visceral pleura helps differentiate this disease from lung abscessation. This is done by observing the line between the affected pleura and the peripheral lung structures, which shows varying degrees of increased echogenicity and thickening.

### 2.5 Pneumothorax

**Figure 4:**
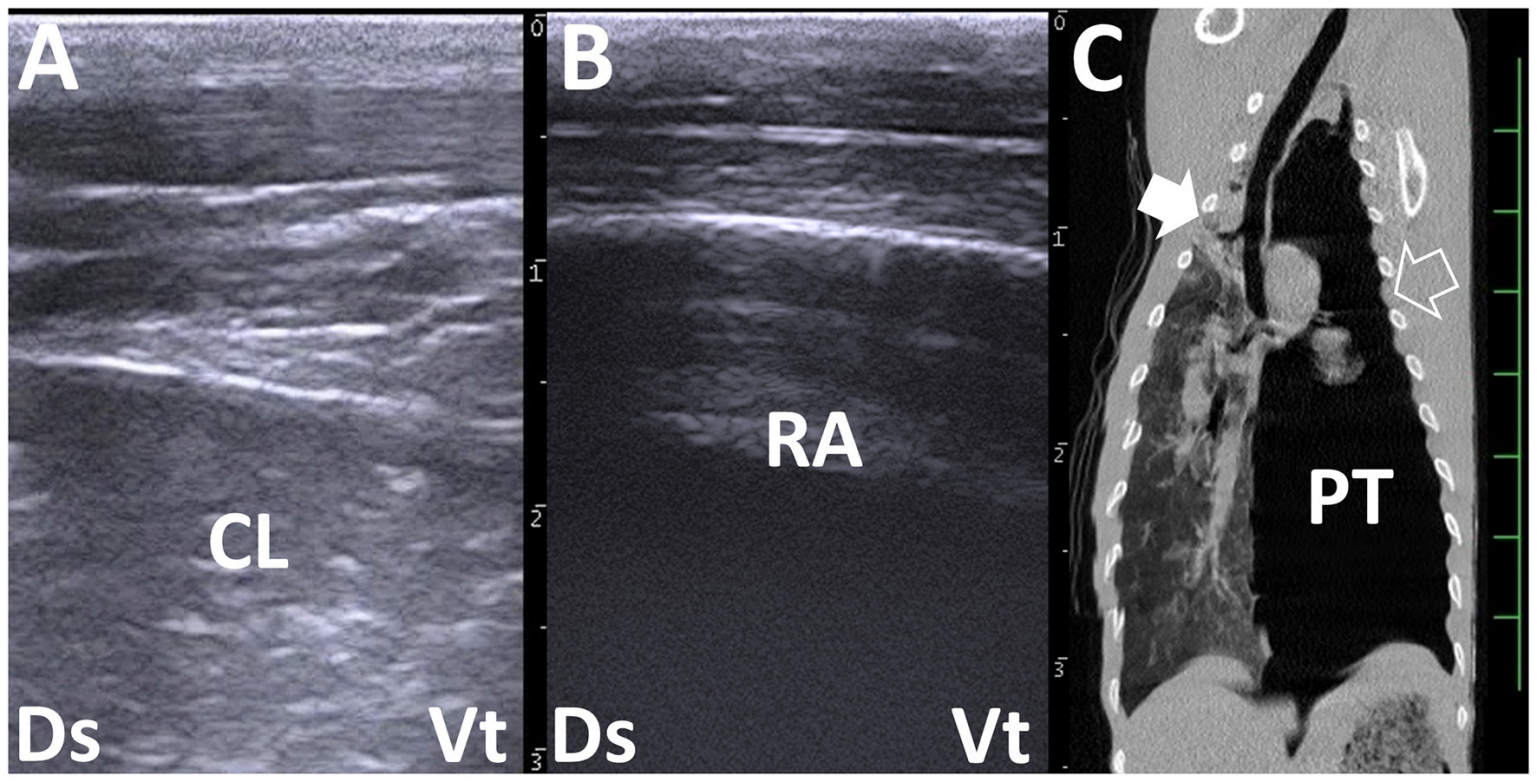
Right and left thoracic ultrasonograms **(A, B)** and computed tomography (CT) **(C)** in a bovine calf with left pneumothorax. **(A)** Increased echogenicity in the lung structures represents consolidated lung (CL). **(B)** Reverberation artifact (RA) is demonstrated extensively within the left dorsal thoracic cavity. No pleural sliding is evident. **(C)** Consolidation in the cranial right lung lobe is demonstrated more deeply than the scanning regions of the right chest (filled arrow). At the scanning region of the left chest (empty arrow), the intrathoracic accumulation of air represents pneumothorax (PT). The scale is 50 mm in the CT image.

Pneumothorax can be a secondary effect of lung infections, with bovine respiratory syncytial virus being the most common pathogen. This virus can cause bronchopneumonia and interstitial pneumonia, accounting for over 80% of bovine cases. Other causes of pneumothorax include trauma, nutritional deficiency, and toxicity (49, 50). Ruptures of the fragile parts of the affected lung structures and emphysematous bullae are the main sources of air outflow into the thoracic cavity, referred to as a closed pneumothorax (49–51). The air-filled thoracic cavity creates a reverberation artifact, which causes the US to miss deep pulmonary consolidation lesions (Figure 4). The risk of missing such lesions in the US can be reduced by identifying typically unilateral involvement, absence of airway sounds at the dorsal region of the affected thoracic cavity through auscultation, and inducing a ping sound from free air within the thoracic cavity through percussion-auscultation (51).

### 2.6 Pericarditis

Traumatic pericarditis is a common pericardial disease in dairy cattle because the reticulum is a common entry point for infection. The strong peristaltic contractions generated by the reticulum can facilitate the perforation of long, sharp foreign bodies from the reticulum itself (52). This can result in injury to the pericardial sac after passing through the peritoneum and diaphragm (7, 46, 52–54). Hematogenous spread of *Pasteurella, Salmonella*, coliform, and anaerobic bacteria is also implicated in cases of pericarditis infection (46). This disease is very rare in sheep and goats, including mycoplasmas, especially *Mycoplasma capricolum* subsp. *Capripneumoniae*, and *Mycoplasma mycoides* subsp. *mycoides* may be the causative agents. It is important to differentiate idiopathic hemorrhagic pericarditis from pericarditis induced by infection, including traumatic pericarditis (7).

The presence of non-inflammatory presternal edema and pulsatile distension of jugular and mammary veins, along with muffled or splashing heart sounds identified through heart auscultation, are noticeable macroscopic findings indicative of pericarditis (46). Thoracic US effectively supports the diagnosis of pericarditis by enabling visualization of the lung, pleura, and heart (46). Pericardial effusion is a specific US finding in pericarditis cases. In buffalos with traumatic pericarditis, pericardial effusion is accompanied by pleural effusion and lung abscessation in ~50 and 13% of cases, respectively (46, 53). The echogenicity of pericardial effusion is commonly hypoechoic to echogenic (7).

**Figure 5:**
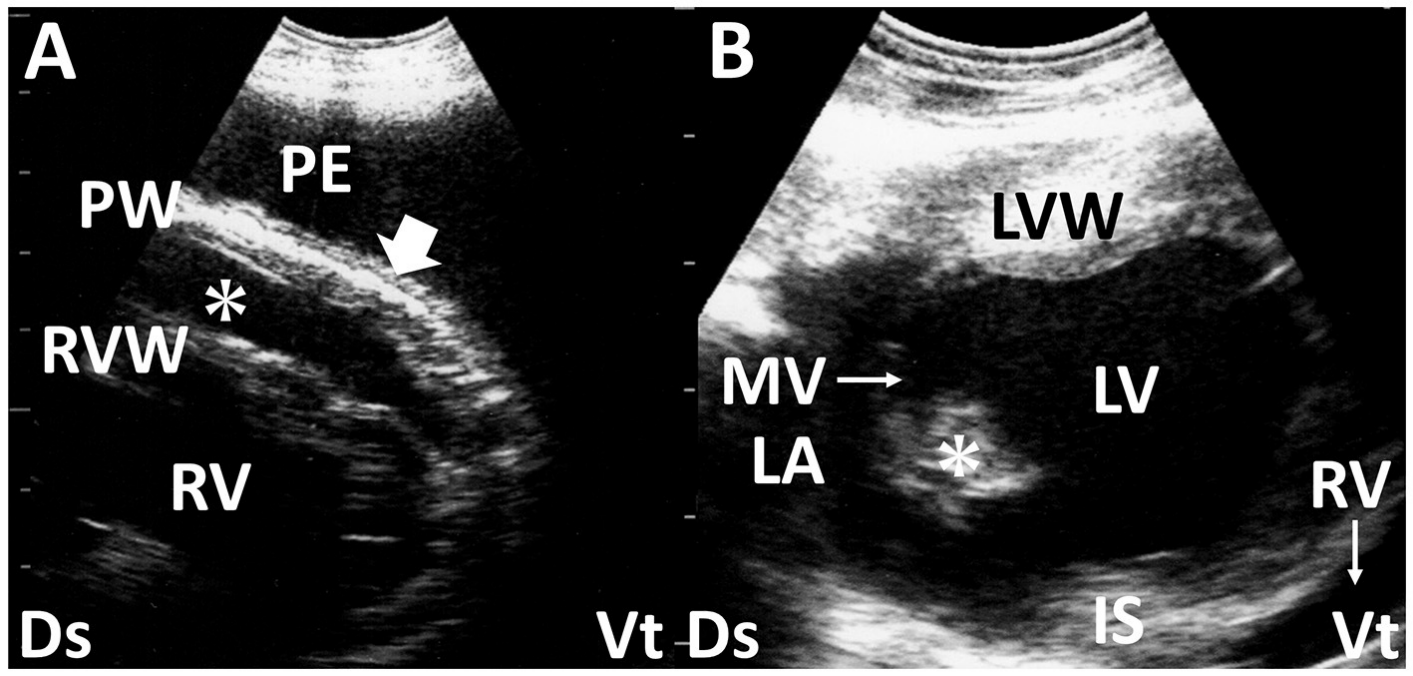
Long-axis echocardiograms show fibrinous pericarditis in sheep **(A)** and vegetation of the mitral valve in deer with endocarditis **(B)**. **(A)** Hypoechoic pericardial effusion (asterisk) is seen within the space between the right ventricular wall (RVW) and the thickened pericardium wall (PW), accompanied by the formation of small, corrugated structures (arrow). A large amount of anechoic pleural effusion (PE) is also evident. **(B)** The echogenic vegetative structure of the mitral valve (MV, asterisk), sized 1.0 × 1.4 cm, is seen in the outflow tract between the left atrium (LA) and left ventricle (LV). IS, interventricular septum; LVW, left ventricular wall; RV, right ventricle; Ds, dorsal; Vt, ventral (61).

Additionally, fibrin threads are formed that protrude from the epicardium and float in the fluid (7, 11, 46, 53) (Figure 5A). In contrast, idiopathic hemorrhagic pericarditis is commonly found in US and is characterized by an accumulation of anechoic pericardial effusion without fibrin clots (7). This distinction helps differentiate it from frequent pericarditis and in the choice of therapeutic options, such as drainage, given the favorable prognosis associated with idiopathic pericarditis (7). Pericardial hemorrhagic effusion caused by epicardial or pericardial lymphohematopoietic neoplasms resulting from bovine leukosis virus (BLV) infection appears as anechoic fluid on US (55). US-guided collection of pericardial effusion is useful for diagnosing this condition based on cytologic examination of the collected fluids, in which lymphocytes are the predominant cellular components (55). Johne's disease induces an accumulation of anechoic pericardial effusion together with pleural effusion in 31% of previous camel cases (56). Pericardial effusions are associated with trypanosomosis in 20% of previous camel cases (57, 58). Combined thoracic and abdominal US is useful for diagnosing trypanosomosis because it can identify accumulations of anechoic to hypoechoic effusions within the peritoneal and pleural spaces and the pericardium (57). Furthermore, continuous use of US during trypanosomosis treatment is effective for evaluating the positive therapeutic effects based on the disappearance of effusions within multiple spaces as indicated by changes in echotexture (58).

### 2.7 Endocarditis

Bacterial endocarditis is the most common endocardial disease in cattle. The condition is defined as an infection of one or more of the endocardial surfaces of the heart. The pathogenesis of this disease is not clearly defined, but chronic active infection leading to sustained or recurrent bacteremia is believed to be a predisposing factor. Echocardiography provides a valuable means of imaging the cardiac chambers and valves in cattle (3, 59, 60). Without the use of echocardiography, it is difficult to diagnose tricuspid, mitral, or pulmonic vegetative endocarditis accurately. Valvular thickening is one of the most sensitive US findings for diagnosing bacterial endocarditis (Figure 5B), as observed in more than 75% of the examined cases (3, 7, 61). The tricuspid valve is the anatomical part most frequently affected (7). US measurement of valvular thickness can support the diagnosis of this disease based on the critical border thresholds of 0.85, 1.27, 1.06, and 0.82 cm in the tricuspid, mitral, aortic, and pulmonary valves, respectively (60). Thrombus formation is sometimes detectable in the affected tricuspid, mitral, and pulmonary valves (3). Additionally, comprehensive US assessment may allow the detection of concurrent problems such as an enlarged liver, distended hepatic veins, and accumulation of effusion within multiple spaces of the pleura, pericardium, and peritoneum (3). In most cases, echocardiography permits an antemortem diagnosis, which can be especially useful in cases with a poor prognosis to avoid ineffective treatment and animal suffering (61).

## 3 Abdomen

### 3.1 Peritonitis

Peritonitis is a focal or diffuse inflammation of the serosal surface of the abdominal viscera or the wall of the abdomen with adhesions as a natural consequence of an inflamed serosa. The condition is common in cattle and rarely clinically identified in sheep or goats (62). The causes of peritonitis are multiple, including uterine tears, ruptured bladder, gastrointestinal perforation, and injury to the peritoneum and diaphragm due to sharp material perforating from the reticulum, referred to as reticuloperitonitis (5, 52, 54). Reticuloperitonitis induces the formation of a reticular abscess and free fluid and fibrin deposits within the abdominal cavity (54). In camels, gastric or intestinal perforation seems to be the frequent cause of peritonitis (63, 64). The pathogen of peritonitis in a llama was reported to be *Streptococcus equi* subsp. *Zooepidemicus*, which may have been transmitted via hematogenous dissemination from pneumonia (64, 65). The bacteria are also the causative agents of peritonitis and pleuropneumonia in dromedary camels (65). Clinical signs of peritonitis include colic, tense abdomen, stomach atony, ileus, weakness, plus or minus fever, diarrhea, painful movement, and recumbency (62). These clinical signs are reported to apply to all species but are non-specific (62).

**Figure 6:**
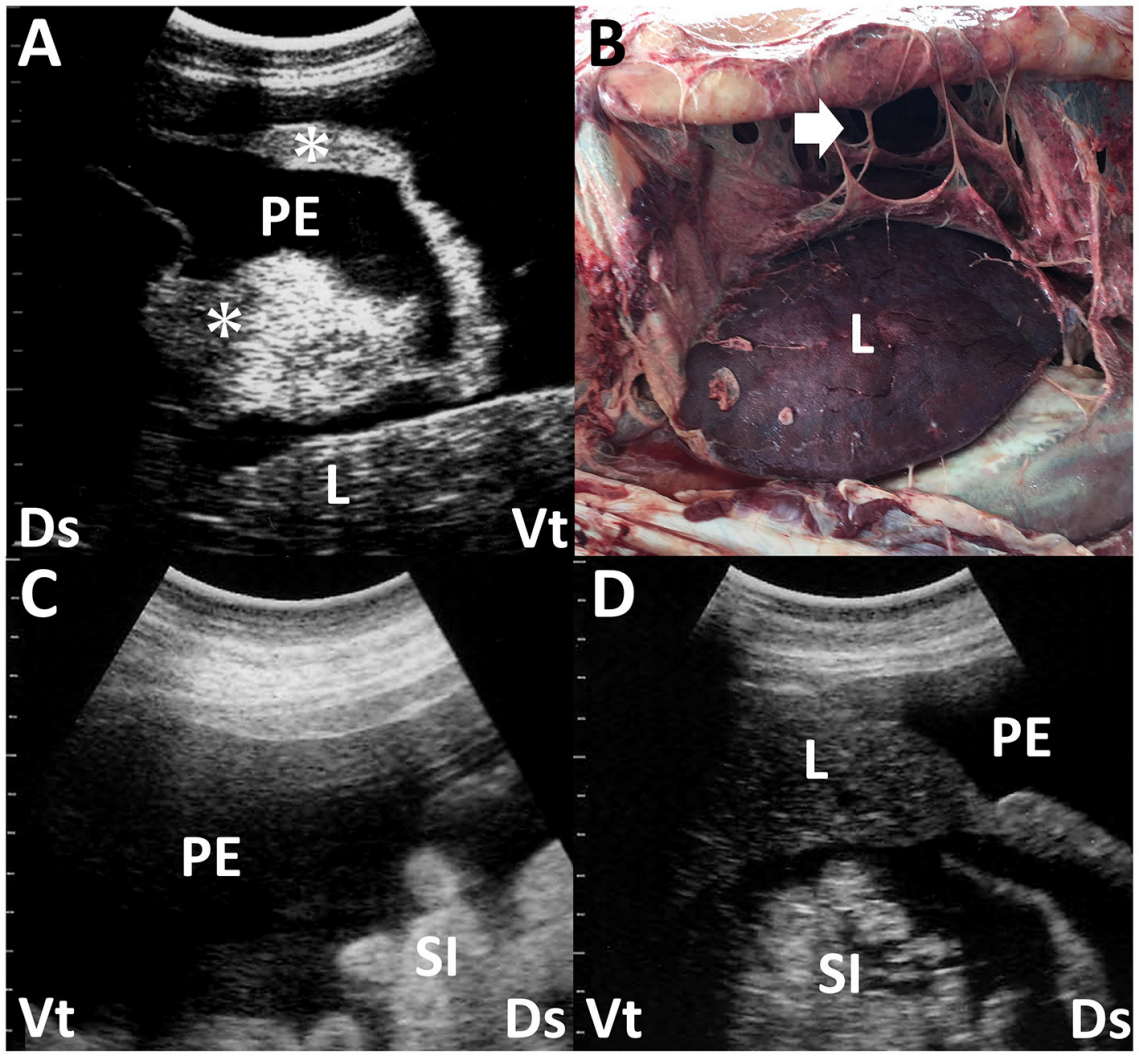
Ultrasonogram **(A)** and macroscopic view **(B)** in a dromedary camel with chronic peritonitis. Ultrasonograms **(C, D)** of a female camel with chronic trypanosomiasis. **(A)** Hypoechoic peritoneal effusion (PE) is seen within the abdominal cavity between the abdominal viscera. The echogenic fibrous strands (asterisk) and liver (L) are imaged floating into the PE. Ds, dorsal; Vt, ventral. **(B)** The formation of the fibrin's sept-like strands (arrow) is evident within the abdominal cavity. Discolored liver structure (L) is also seen (63). **(C, D)** Hypoechoic peritoneal effusions (PE) are seen within the abdominal cavity, in which the small intestines (SI) and liver (L) are floating. Ds, dorsal; Vt, ventral (58).

US of the peritoneum has been cited as the best method to assess the extent of peritoneal reaction/abscessation in ruminants (17, 53, 57, 62, 63). Intraabdominal abscesses are ultrasonographically characterized by an echogenic capsular mass of varying thickness enveloping the hypoechoic to echogenic contents, sometimes generating acoustic shadowing (17, 18). Intraabdominal accumulations of the peritoneal effusions and abscessation associated with reticuloperitonitis can be identified with US scanning at the paramedian ventral areas within the 6th to 12th intercostal spaces (9, 52). Inflammatory reaction within the abdomen causes the creation of fibrins, represented as hypoechoic to hyperechoic strands, floating into the effusion or forming as septa-like structures, including effusions within the spaces between the peritoneum, greater omentum, and viscera such as the intestines, liver, kidneys, rumen, and spleen (9, 17, 54, 63, 66) (Figures 6A, B). In 50% of camel cases, the formation of fibrin strands into hypoechoic effusions that accumulate between intestinal loops was observed by the US as a complication of intestinal obstruction despite the absence of fibrin strands in 33% of the camel cases (57). Small amounts of effusion can be commonly demonstrated using the US but may be difficult to detect within the adhered mass of the ruptured small intestines as the effusion source (66, 67).

**Figure 7:**
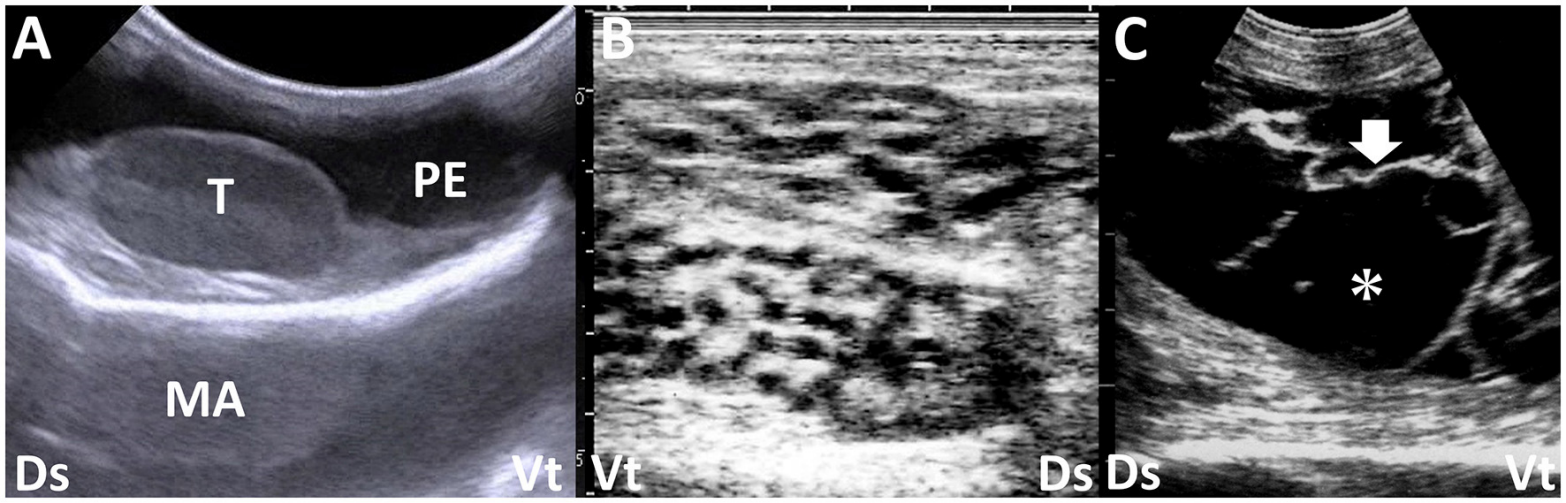
Ultrasonograms of the swollen scrotums in a calf with peritoneal mesothelioma **(A)** and two bucks with epididymitis **(B)** and periorchitis **(C)** caused by infections with *Brucella melitensis*. **(A)** Anechoic peritoneal effusion (PE) accumulates around the normal structure of the testis (T) within the scrotum. MA, mirror image artifact (image courtesy of Dr. Yasuhiro Morita, who belongs to Kyushu University). **(B)** Honeycomb-like structures of the epididymis are seen. **(C)** Echogenic fibrous strands (arrow) are seen on the tunics surrounding the atrophied testis and floating within anechoic effusion (asterisk). Dr, dorsal; Vt, ventral.

Peritoneal effusion is one of the most sensitive indicators of peritonitis in the US, but it is nonspecific because other diseases can also cause peritoneal effusion (54). Peritonitis can induce local or general accumulations of exudates with varying echogenicity depending on the cell counts and amount of fibrin (66). Anechoic fluids in the US in non-inflammatory abdominal effusions are mainly caused by increased intravascular hydrostatic pressure and/or decreased intravascular colloid osmotic pressure (66). Types of peritoneal effusion associated with fascioliasis vary (23). They include the accumulation of modified transudates or exudates in secondary peritonitis and transudates caused by hypoalbuminemia, leading to decreased intravascular oncotic pressure (66). Trypanosomiasis, caused by *Trypanosoma evansi*, results in Surra, a severe protozoal disease affecting camels in the Middle East, Africa, and Asia (58, 68, 69). Common US characteristics of this disease include massive intraabdominal accumulations of anechoic or hypoechoic fluids (58) (Figures 6C, D). Identification of peritoneal effusion via the US may indicate chronic trypanosomiasis, presenting clinically as chronic weight loss and subcutaneous edema (6, 57, 58, 69). The uroperitoneum typically manifests as an anechoic peritoneal effusion, which is distinct from the US findings of peritonitis (11, 19, 28, 34, 57, 70–72). However, the retention of intraabdominal urine can frequently lead to secondary peritonitis, increasing the echogenicity of peritoneal fluids, including strands of hyperechoic fibrin (28). Peritoneal mesotheliomas often present as anechoic patterns of peritoneal effusion in the US (73). This generates transudates with fewer cellular components throughout the abdominal cavity, sometimes leading to anechoic fluid accumulation in the scrotum (73). It is necessary to distinguish between scrotal enlargement associated with massive peritoneal effusion seen in the US vs. enlargement due to orchitis and/or epididymitis, which are mostly associated with brucellosis in rams and bucks (74) (Figure 7). Although the echogenicity of peritoneal effusion is an effective indicator for differentiation between peritonitis and other diseases, it is not diagnostic. Therefore, US-guided abdominocentesis effectively collects peritoneal effusion because laboratory examination is highly sensitive in diagnosing peritonitis (18, 54, 73).

### 3.2 Liver abscessation

**Figure 8:**
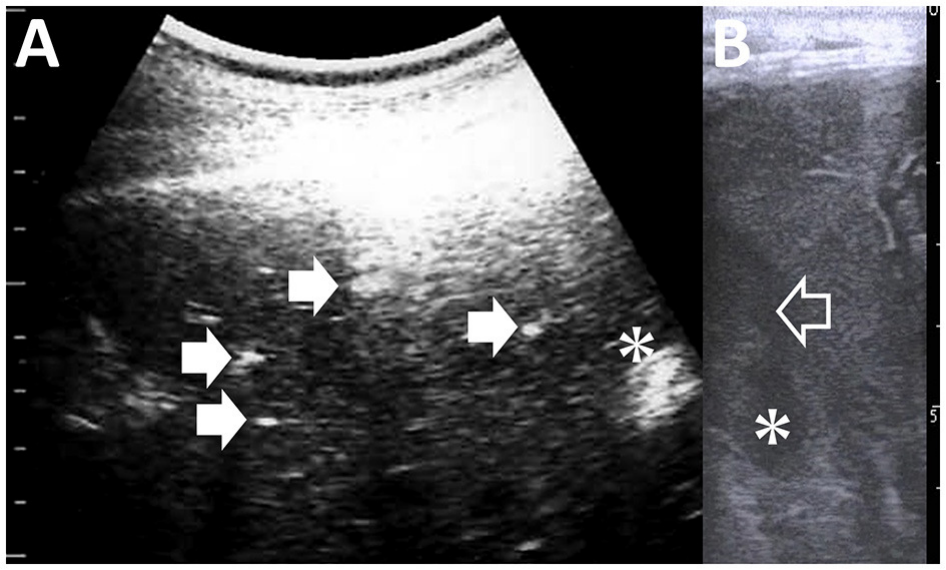
Ultrasonograms of liver abscess in a camel **(A)** and in a calf with umbilical vein infection **(B)**. **(A)** A 1-cm-sized abscess (asterisk) is characterized as the capsular mass enveloping the heterogeneous echogenic contents. Arrows in the image point to the calcified bile ducts. **(B)** Anechoic contents are evident within the lumen of the umbilical vein (empty arrow) and the cavity of a 2-cm-sized abscess (asterisk).

Liver abscessation has a major economic impact on the feedlot industry due to liver condemnation and reduced animal performance and carcass yield. It can occur at any age and in any type of cattle, contributing to the greatest economic loss in grain-fed cattle (75). This disease is often diagnosed incidentally during postmortem examination due to difficulty detecting its clinical signs, even when the animals have hundreds of small abscesses or several large abscesses (20). In a retrospective study on isolates from camel liver abscesses, *Staphylococcus* spp., *Corynebacterium* spp., and *Streptococcus* spp. were the predominant pathogens (76). US of liver abscesses can appear as single or multiple formation of masses sized between 3 and 20 cm (11, 77, 78). These US findings appear to change depending on the entry of infection, inflammatory reaction, and chronicity (77, 78). Liver abscesses seldom infiltrate the peripheral liver parenchyma, making it easy to distinguish the lesion's border by US. However, the formation of septa-causing chambers in the mass may indicate partial destruction of liver structures, possibly related to the chronicity of liver abscessation (77). The echogenicity of abscess contents varies from anechoic to hyperechoic (11, 75) (Figure 8).

Additionally, abscess contents can appear heterogeneously or homogeneously, although long-standing liver abscesses tend to have homogeneous contents (77). US is useful for guiding centesis procedures to differentiate liver cysts from tumors and for drainage during treatment (77). US can also provide significant evidence for choosing therapeutic options and assessing prognosis. Identification of multiple liver abscesses by the US indicates an unfavorable outcome, often associated with serious conditions such as sepsis and local spread from infectious foci within the umbilical remnants (77, 78). Evaluating the intrahepatic distribution of lesions by US may help decide whether surgical therapy is necessary for liver abscesses, even if they affect multiple areas (78). This approach was successful in a recent case of liver lobectomy for a calf with multiple liver abscesses (78).

### 3.3 Fascioliasis

Fascioliasis is caused by the infestation of *Fasciola hepatica* into the hepatobiliary system. The acute phase corresponds to the intrahepatic migration of immature parasites through the intestinal mucosa, known as the hepatic stage (11, 79). The chronic phase occurs when adult flukes establish themselves in the biliary ducts, referred to as the biliary stage (79). During the biliary stage, biliary obstruction develops, and plasma protein leaks across the epithelium, leading to hypoalbuminemia. There is also whole blood loss due to the feeding activities of the flukes, exacerbating anemia and hypoalbuminemia (80). As adult worms increase, the likelihood of developing liver fibrosis increases (81). The fibrotic response of the liver to fluke-induced damage varies among hosts and may partly account for differing species' susceptibilities (82).

**Figure 9:**
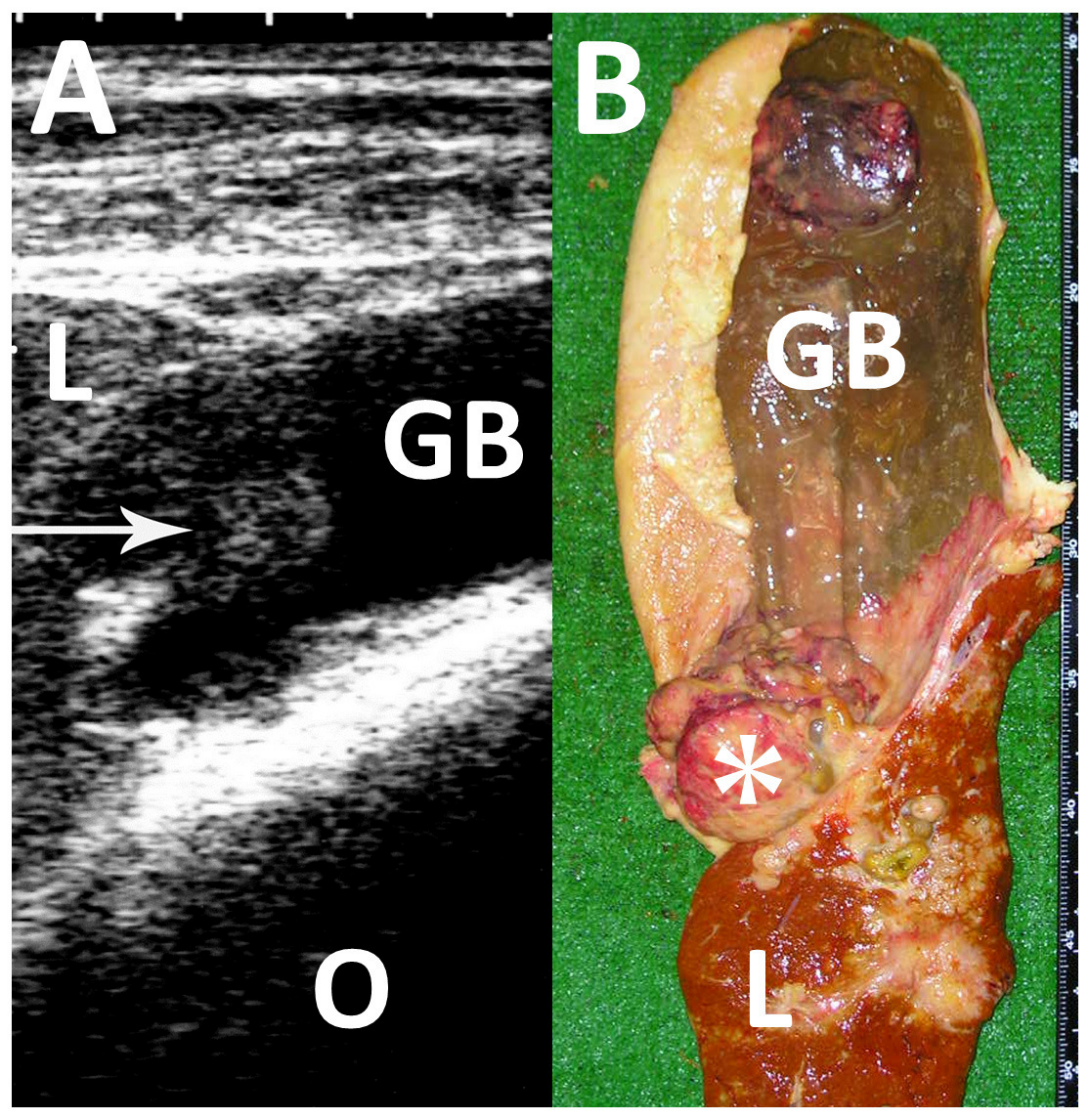
Ultrasonogram **(A)** and macroscopic view **(B)** in a Japanese black cow with fascioliasis. **(A)** An echogenic mass (arrow) is seen within the gallbladder (GB). L, liver; O, Omasum. **(B)** A 5-cm cauliflower-like mass (asterisk) is seen between the discolored liver structure (L) and gallbladder (GB) (80).

Based on previous experimental studies using rabbit and ovine models, the US characteristics of fascioliasis may change depending on the development of hepatic and biliary stages (79, 83). These studies initially identified the intrahepatic distribution of hypoechoic to hyperechoic lesions, followed by dilated, tortuous biliary ducts containing active echogenic parasites in their lumens during 7–8 weeks and 9–10 weeks after *Fasciola hepatica* infection, respectively (79, 83). However, naturally infected animals may exhibit a mixture of pathological changes corresponding to the hepatic and biliary stages due to the time lag between *Fasciola hepatica* infection and symptom onset (79). Cattle and buffaloes naturally affected by fascioliasis show heterogeneous echotexture of liver parenchyma in US, with scattered hyperechoic spots, bile duct enlargement, and gallbladder wall thickening (11, 23, 80) (Figure 9). In cases where fascioliasis causes severe cholangiohepatitis and inflammatory liver fibrosis, the liver parenchyma appears heterogeneous. Chronic fascioliasis is the most common cause of bile duct calcification, which, in the US, generates acoustic shadowing distal to the hyperechoic walls of affected bile ducts (11, 23, 77, 80). This manifests as ring-like or tube-like echotexture in US, observed in cross-sectional or longitudinal views (77, 80). In addition to identifying liver lesions, US helps guide cholecystocentesis for detecting liver fluke eggs and facilitates cytological and bacteriological examinations of bile fluids aspirated via a spinal needle introduced percutaneously (77).

### 3.4 Hydatid cysts

**Figure 10:**
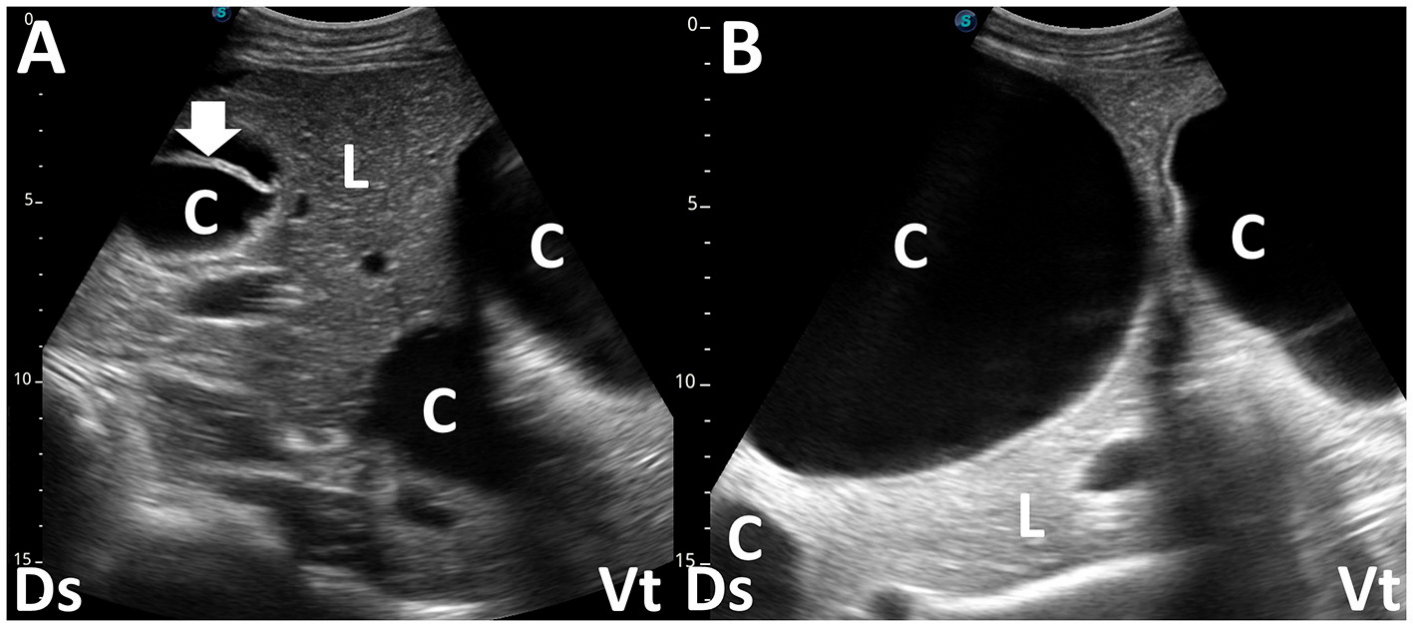
Ultrasonograms **(A, B)** in a female camel with liver hydatidosis. **(A)** Three cystic masses (C) enveloping anechoic fluids are evident within the compressed liver parenchymas (L). Thickening in the wall of one mass corresponds to an early daughter cyst (arrow). **(B)** Two large and one small cystic mass (C) are seen within the liver (L). Ds, dorsal; Vt, ventral (84).

The adult tapeworm, *Echinococcus granulosus*, is found in the intestines of carnivores, particularly dogs (84). Eggs are passed in the feces and ingested by sheep, goats, other ungulates, or humans (84). In these intermediate hosts, ingested eggs release oncospheres that enter intestinal venules or lacteals and migrate to the liver or lungs via the circulatory system. The metacestode stage, known as the hydatid cyst, develops in these organs over several months. Hydatid cysts typically average 5–10 cm in diameter, contain a yellowish, serum-like fluid, and may have a granular inner wall with multiple brood capsules (11, 26, 48). Hydatid “sand,” which is an accumulation of detached brood capsules, may be observed in the cyst fluid. US examination of sheep with hydatid cysts shows multiple cysts with anechoic content in the abdomen (26, 85). An elongated echogenic structure corresponding to the scolex is typically seen in the center of the cyst. Cysts are most commonly found in the liver, lungs, and spleen (84) (Figure 10). US detection of liver hydatid cysts accounts for 9.2 and 2.5% of examined ovine and caprine cases, respectively (86). US sensitivity and specificity for detecting these lesions at necropsy are 54.4 and 97.6%, respectively (85). The classification criteria for cystic echinococcosis in veterinary fields in the US align with those of the World Health Organization (79, 84, 87). These criteria categorize cystic disease into five types (79, 87). Active and fertile cysts are demonstrated by the intrahepatic formation of rounded, unilocular, anechoic, well-defined nodules with or without septa in the US (79, 87). The formation of the mass appearing with an irregular contour and variable echogenicity indicates the inactive stage in cystic echinococcosis (79, 87).

### 3.5 Cysticercosis

**Figure 11:**
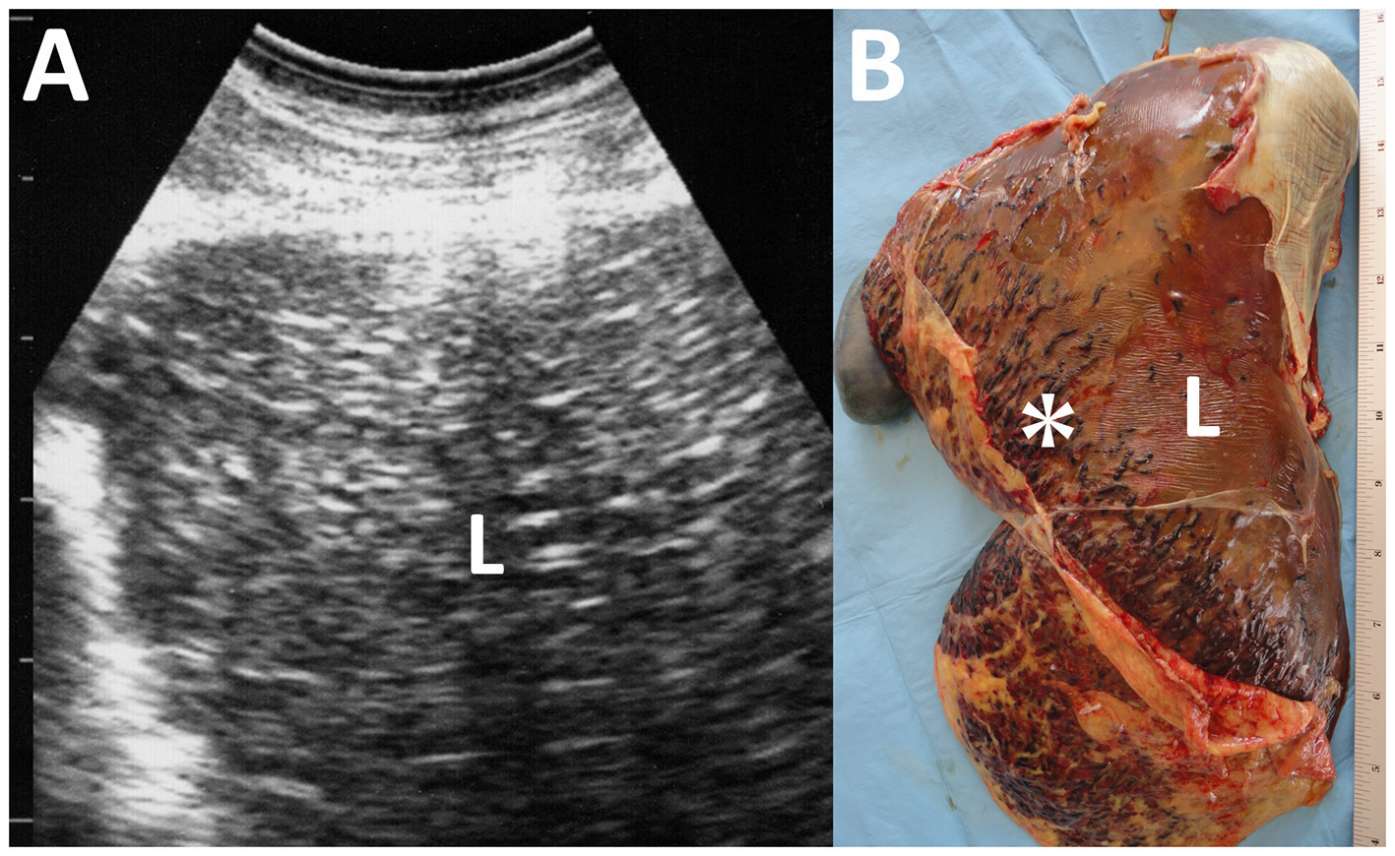
Ultrasonogram **(A)** and macroscopic view **(B)** of cysticercosis in the liver of an ovine case. **(A)** The liver parenchyma (L) is demonstrated as heterogeneously hyperechoic. **(B)** Blood-filled tracts (asterisk) are seen within the swollen liver (L) due to the migration of *Cysticercus tenuicollis* (the embryos of *Taenia hydatigena*).

Ovine visceral metacestodiasis is caused by the migration of *Cysticercus tenuicollis*, the intermediate stage of *Taenia hydatigena*, found in the intestines of dogs, coyotes, wolves, and other carnivores, to the liver and lung tissue of intermediate hosts such as sheep, goats, cattle, pigs, and squirrels (88). The adult tapeworm of this larval form is the canine tapeworm *Taenia hydatigena*. Onchospheres released from eggs penetrate the intestine and travel via the portal vein to various tissues, especially the liver, omentum, mesentery, and peritoneum. Migration through the liver causes hemorrhagic tracks, and on reaching the liver surface, the larva develops into a thin-walled, fluid-filled bladder (89). Alternatively, it may degenerate, calcify, or occasionally predispose to black disease (89). The US of the affected liver typically shows a diffuse hyperechoic pattern in the liver parenchyma (88). Acute cysticercosis is characterized by the presence of tubular, red, blood-filled tracts 2–4 mm in diameter within the liver parenchyma, where cysts may also be present (Figure 11). Similar US findings are common in the mesentery, omentum, and serosal surface of peritoneal viscera.

### 3.6 Pyelonephritis

**Figure 12:**
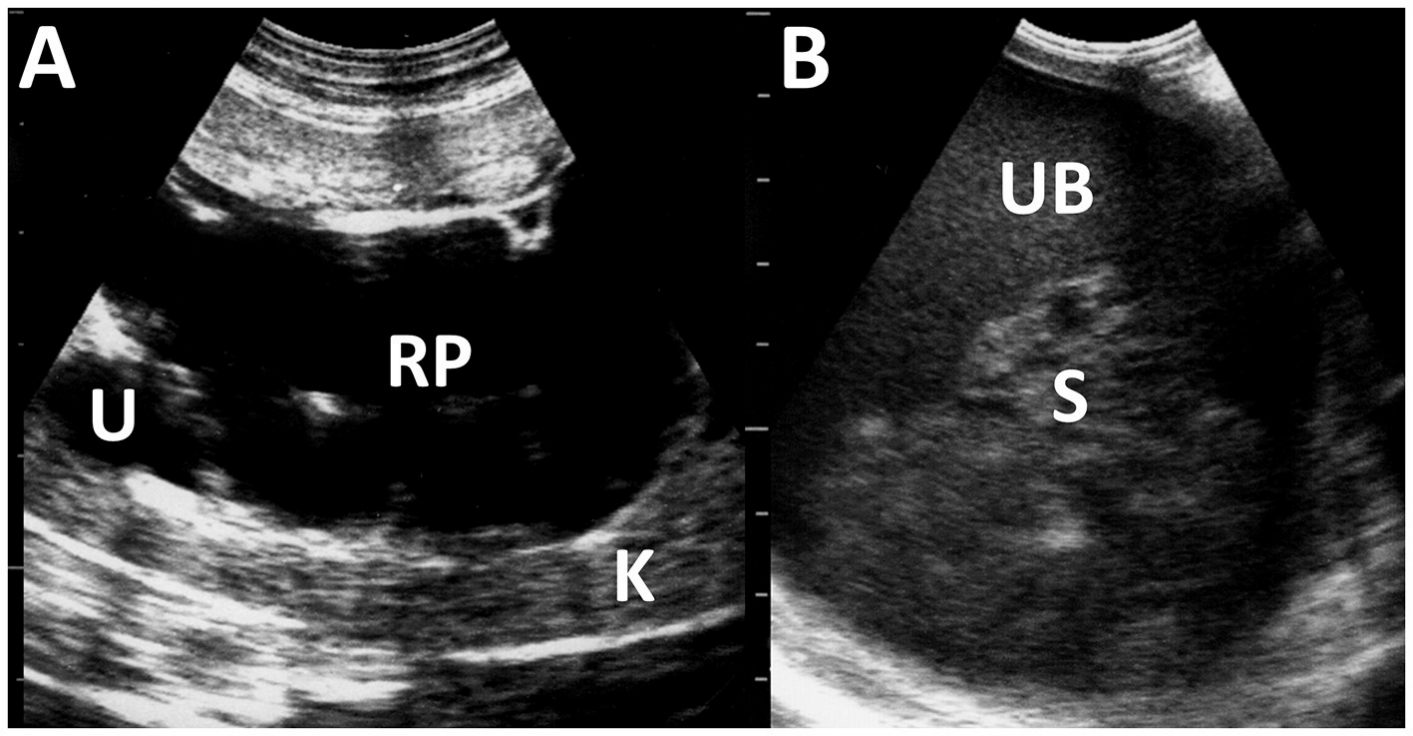
Ultrasonograms of pyelonephritis in the kidney of a caprine case **(A)** and the urinary bladder of an ovine case **(B)**. **(A)** The affected kidney (K) has loss of normal echotexture of its parenchyma and increased echogenicity. Dilated renal pelvis (RP) and ureter (U) include anechoic urine. **(B)** The echogenicity of the urine is increased within the lumen of the urinary bladder (UB). Echogenic sediments (S) of cell debris, pus, and red blood cells are also evident.

Pyelonephritis is an infectious renal disease caused by local or systemic infection by less common isolates, including *Escherichia coli, Staphylococcus* spp., *Streptococcus* spp., *Enterococcus* spp., *Klebsiella* spp. *Trueperella pyogenes* and *Pseudomonas* spp. (29, 31, 32, 90). According to previous abattoir survey examinations, the prevalence of pyelonephritis may be lower in sheep and goats than in cattle, accounting for between 0.9 and 3.5% (29). Common US characteristics of the affected kidney include a dilated and deformed renal sinus and an unclear echogenicity border between the renal cortex and medulla (6, 27–29, 31, 32, 54, 91) (Figure 12A). Typically, the dilated ureter appears at the location of the renal pelvis outlet. It contains hyperechoic purulent debris, red blood cells, and pus (27, 28, 91). When using US to identify dilation in the renal pelvis and ureter, it is essential to differentiate it from hydronephrosis, which is characterized by dilated renal pelvis and pressure atrophy of the renal parenchyma (30, 71, 72, 90). Echogenic foci generate acoustic shadowing, which is evident if the exudates accumulate and crystal deposits are present within the renal parenchyma (27). The echotexture of the affected kidney shows renal abscessation, characterized by a capsular mass enveloped by a hyperechoic capsular wall and hypoechoic content (30, 32). Regarding the US appearance of other infectious renal diseases, BVD infection can cause glomerulonephritis, appearing as hypoechoic medullary pyramids (28). This appearance contrasts with the increased echogenicity of the renal cortex seen in dilated renal parenchyma, which subsequently decreases in size during the chronic phase (28). Embolic nephritis can occur secondary to septicemia (28). Some affected animals develop endocarditis secondary to systemic bacterial infection (28). US can identify hypoechoic foci formation in the renal cortex, although these lesions are often small and difficult to detect (28). The US is useful for guiding percutaneous centesis of the kidney for aspiration of purulent materials and biopsy, allowing differentiation between various renal diseases (30).

### 3.7 Cystitis

Cystitis is often caused by bacterial infection, resulting in urinary bladder inflammation (28). The US of cystitis identifies diffuse thickening in the bladder walls due to thickened and corrugated mucosa (28, 71). The affected urinary bladder includes multiple echogenic fluid contents swirling within the lumen (28). In some animals with pyelonephritis, the fluid contents within the urinary bladder show increased heterogeneously echogenicity due to the outflow of purulent materials into the lumen (27, 28) (Figure 12B). Therefore, if there is a change in urine echogenicity, the bladder and kidney should be scanned together using US.

### 3.8 Enteritis

**Figure 13:**
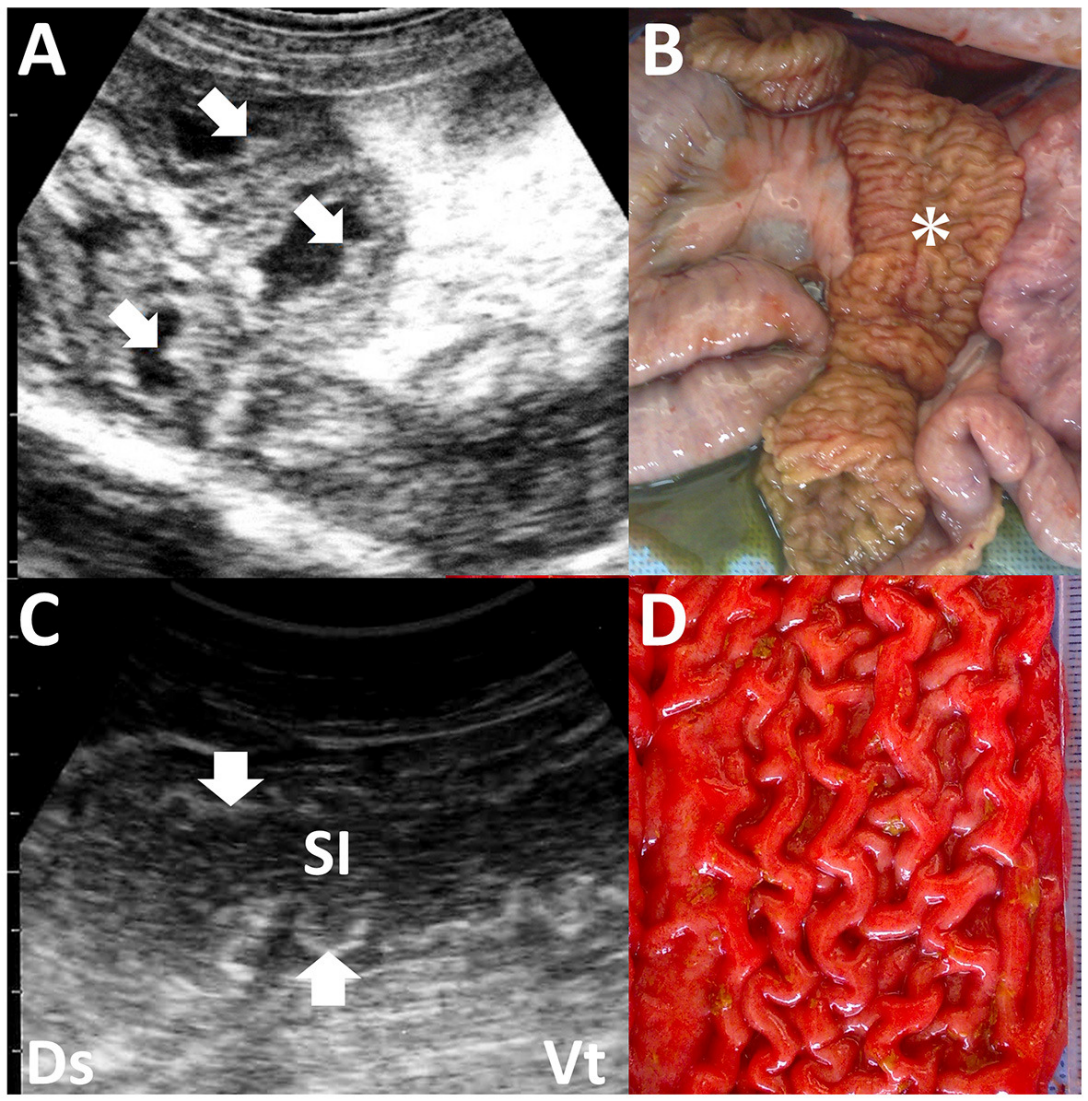
Ultrasonograms and macroscopic views of Johne's disease in a caprine case **(A, B)** and camel's case **(C, D)**. **(A)** The corrugated intestinal mucosa (arrows) is visible on the thickened intestinal walls. **(B)** Corrugated changes (asterisk) in the intestinal mucosa are seen extensively (94). **(C)** The longitudinal section of the corrugated intestinal mucosa appears as wavelike lines (arrows) within the dilated lumen of the affected small intestine (SI). **(D)** Corrugation of the intestinal mucosa is evident on the whole surface of the affected intestine (56).

Johne's disease is caused by *Mycobacterium avium* subsp. *paratuberculosis* infection, also known as paratuberculosis. The disease is characterized by chronic, contagious enteritis, leading to chronic wasting and fatal development of persistent diarrhea, ultimately resulting in death (56, 92–96). This organism may cause Crohn's disease in humans (97). Therefore, strict isolation and eradication of infected animals are required. The clinical use of US is useful for isolating suspected animals until confirmatory laboratory tests allow a conclusive diagnosis of Johne's disease. US commonly identifies the thickening of the intestinal walls in most affected ruminants, such as cattle, camels, goats, and sheep (56, 94, 98). In caprine cases with Johne's disease, US identifies thickened intestinal walls measuring >2.0 mm, typically including corrugation of the intestinal mucosa (94) (Figures 13A, B). In camels affected with Johne's disease, thickening and corrugation in the intestinal walls are also common US characteristics, identified in 84% of previous cases (56) (Figures 13C, D). The severity of intestinal wall thickness is classified as mild, moderate, or severe based on US measurements (56). Specifically, the values for mild, moderate, and severe are 6.8, 12.8, and 17.5 mm, respectively, compared to the normal value of 3.6 mm (56). In 95% of camel cases, the thickening and corrugation of the intestinal walls identified by US can represent macroscopic changes, such as increased sizes in the folds of the mucous membrane within the lumens of the affected intestinal loops (56, 94, 95). Other US findings include increased hepatic echogenicity, omental edema, and peritoneal, pleural, and pericardial effusions in affected caprine, ovine, and camel cases (6, 56, 94). Additionally, these cases have enlargement of the mesenteric lymph nodes demonstrated ultrasonographically as the hypoechoic cortex and hyperechoic medulla (6, 94). The enlarged lymph nodes are identified as another US finding in which an echogenic capsule envelops anechoic, echogenic, or heterogeneous parenchymas (6, 56). When US identifies intraabdominal swollen masses, it is necessary to differentiate lymphadenopathy associated with infectious gastrointestinal diseases such as Johne's disease from intraabdominal abscess, enzootic bovine leukosis, and fat necrosis (99). BLV-associated lymphadenopathy and fat necrosis are commonly demonstrated as heterogeneous hyperechoic and hypoechoic mass lesions, respectively (4, 99). US helps guide the biopsy of intraabdominal masses, aiding the differential diagnosis (4, 91).

Eosinophilic enteritis is an inflammatory bowel disease caused by infiltration of eosinophils. It occurs idiopathically or secondary to parasitism, among other causes, such as drug reactions, systemic eosinophilic syndrome, and malignancy in ruminants (100). Thickening in the intestinal walls, with a dilated lumen filled with fluid, is a common US characteristic of this disease, representing inflammation of the intestinal mucosa, lamina propria, and submucosa (100).

Hemorrhagic bowel syndrome is an acute necrohemorrhagic enteritis, with *Clostridium perfringens* infection considered the most likely cause (101). The accumulation of heterogeneously echogenic masses of blood clots in the lumen of the small intestine is indicative of this disease, as identified by the US in 19% of affected animals (101, 102). Thickening in the intestinal walls is evident in only 10% of cases (102).

### 3.9 Umbilical remnant infection

Infection in the external stump, or omphalitis, is a macroscopically detectable umbilical infection that induces various degrees of umbilical swelling (103, 104). The umbilical infection can transfer pathogens into the lumens of the internal umbilical remnants, including the umbilical vein, umbilical artery, and urachus, resulting in omphalophlebitis, omphaloarteritis, and urachitis, respectively (33, 104). Palpation cannot always detect umbilical remnant infections despite helping diagnose omphalitis (103). US is an effective imaging tool that provides high-quality images for evaluating intraabdominal involvement during percutaneous scanning (21).

**Figure 14:**
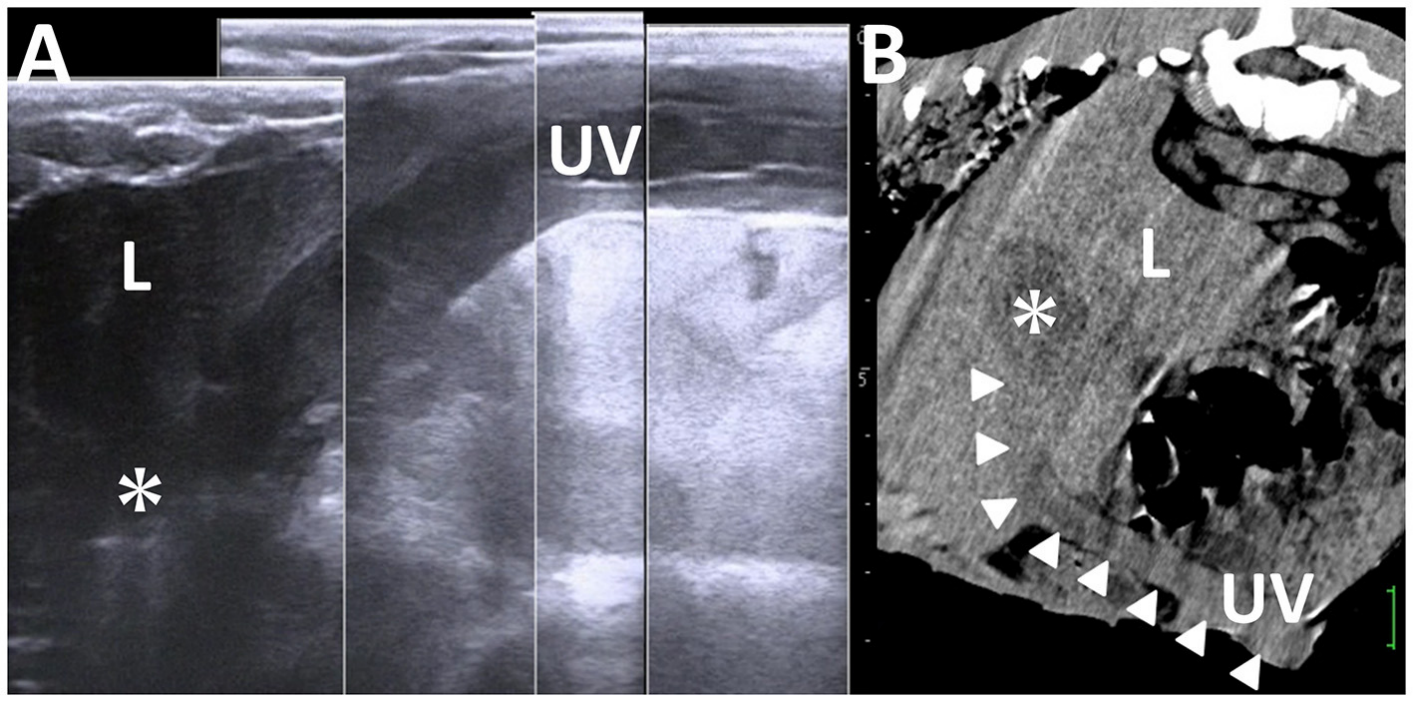
Ultrasonogram **(A)** and computed tomography **(B)** of liver abscessation associated with umbilical vein infection in a calf. **(A)** The tubular structure of the umbilical vein (UV), including anechoic contents, runs between the umbilicus and the liver (L) and ends at the capsulated mass (asterisk) within the L. **(B)** The umbilical vein (UV) is demonstrated as the entry of infection (arrowheads) from the umbilicus to the capsulated mass (asterisk) within the liver (L). The scale is 25 mm in the CT image.

**Figure 15:**
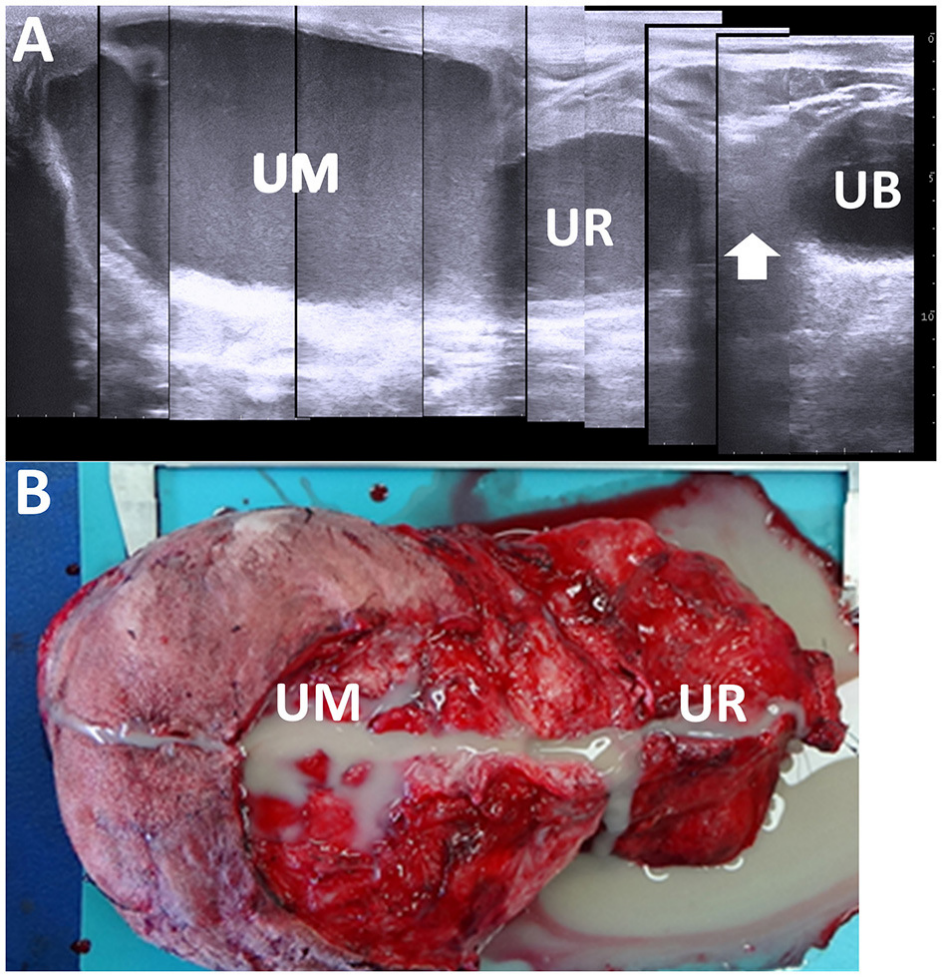
Ultrasonogram **(A)** and macroscopic view **(B)** in a calf with abscessation extending between the umbilicus and urachus. **(A)** There are echogenic contents within the extended lumens of the umbilicus (UM) and urachus (UR), sized at ~10 and 5 cm, respectively. The cord-like structure (arrow) is seen between the extended UR and the urinary bladder (UB). **(B)** The extended mass of the umbilicus (UM) and urachus (UR) enveloping the pus is removed surgically after suturing and cutting at the part of the cord-like structure.

In 50% of the healthy calves aged 3 weeks old, the US detects the umbilical vein as a round anechoic to hypoechoic structure within the abdominal cavity (33, 103). An abnormality in retraction after umbilical cord break is considered if the full length of the umbilical vein is identified in the US at this age (33). US can provide evidence to evaluate the association between the umbilical vein and liver abscess formation (21, 33). During US, a urachal abscess can be identified as a tubular structure with varying wall thickness and echogenic materials. This structure typically enters the liver when scanning from the midline of the ventral abdominal surface toward the right side and moving cranially from the umbilicus (19, 21, 33) (Figure 14). The urachus is a common route for the intraabdominal spread of umbilical infection into the urinary bladder in younger animals, where it can be identified as a tubular structure present at birth (33). The urachal abscess appears in US as an accumulation of echogenic contents with or without small hyperechoic deposits within the extended lumen of the tubular structure (19, 105) (Figure 15). The degree of extension of the affected urachal lumen, including the purulent materials, is a significant factor in surgical decision-making (105). The spread of infection between the umbilical cord and urinary bladder via the umbilical vein can be assessed by the flow of echogenic contents within the umbilical vein toward the lumen of the urinary bladder (33, 70, 105). The umbilical artery may serve as an entry point for systemic infection via the aorta (104). The umbilical artery is detectable when applying US to healthy calves up to 1 month old, but its diameter decreases with age (104). In the US of omphaloarteritis, the diameters of affected umbilical arteries are observed to remain stable but subsequently increase with growth (104). However, the US diagnosis of omphaloarteritis is more difficult than that of omphalophlebitis and urachitis (21).

## 4 Utility of US compared with the other imaging modalities

Thoracic radiography can distinguish lower lung field density, providing good contrast with the hyperdense contours of the heart and diaphragm located in the central and caudal aspect of the thoracic cavity. Abnormal shape and contour of the heart and diaphragm are radiographic evidence suggestive of pericarditis, including an unclear diaphragm line, gas opacity overlapping the caudal contour of the heart, and an enlarged dorso-caudal cardiac silhouette (10). However, radiography has a lower sensitivity to detect this disease, especially in cattle, compared with the use of US (10). On thoracic radiographs, the soft tissue opacity of the lung becomes a good background to highlight various types of pulmonary lesions, such as lung cysts and abscesses appearing as round to oval radiopaque masses, depending on their sizes (48). Radiography can also identify specific cavitary lung lesions, such as gas-forming bacteria-induced lung abscesses, including gas-fluid interfaces and gas-filled masses of emphysematous bullae (36). The radiographic detection sensitivities of lung abscesses and emphysematous bullae are 30 and 38%, respectively, whereas pneumothorax has a sensitivity of 0% (106). This indicates that pneumothorax may be difficult to diagnose using thoracic radiography, identical to thoracic US based on Figure 4. Thoracic radiography helps distinguish between three types of pneumonia classified as alveolar, interstitial, and bronchial patterns (106). However, a mixture of these three patterns is common in ruminants with pneumonia, making it difficult to differentiate among them using radiographs (106). When thoracic radiography reveals hypodense structures of air-filled bronchi enhanced by the diffuse, hyperdense changes of the affected lung structures, referred to as an air bronchogram, this radiographic sign is among the most useful evidence to confirm consolidation (36, 107). This radiographic abnormality mostly appears within the cranioventral lung regions in ruminants (15, 108). However, this region normally overlaps with the opacity of the forelimbs on lateral thoracic radiographs taken in a standing position (108–110). Physical or drug restraints of the examined animals are required to place them in a lateral recumbent position to obtain optimal lateral thoracic radiographs, with their forelimbs pulled cranially to sufficiently evaluate cranioventral lung regions (108, 110). However, forced examination may lead to the progression of weakness and exacerbation of pneumonia in the examined animals (107). Therefore, new radiographic techniques have been developed to provide good views of cranioventral lung fields, such as the two-legged technique, where the examined calf's body is held up with its forelimbs pulled cranially (108), and the three-legged technique, where the animal stands on one forelimb and both hindlimbs while lifting the opposite forelimb for lateral thoracic radiography (110). These radiographic techniques may take several attempts due to unexpected movement and faster respiratory motion in struggling animals, making this examination very stressful for both examiners and examined animals (36).

Regarding observation of the cranioventral lung field, thoracic US is not superior to thoracic radiography (110). In the thoracic US, visualizing thoracic regions between the 1st and 3rd intercostal spaces is very difficult due to the scanning operation to maneuver the transducer into the axillary space (12, 41, 110). Although a previous report described a bovine thoracic US technique that extended scanning areas to the 1st to 2nd intercostal spaces, this technique seems to cause great discomfort in the examined animals. Additionally, this technique may be applicable only to very young calves estimated to be under 12 weeks of age (12, 110). Therefore, despite being more routinely available, thoracic UA cannot completely replace thoracic radiography as a diagnostic technique (110).

The radiopacity on abdominal radiography is mostly homogeneous in the abdominal viscera themselves. In contrast, heterogeneous radiodense structures of the ingesta and radiolucent gas are visible within the lumens of gastrointestinal tracts. Differences in radiodensity between abdominal viscera, omentum, and intraabdominal fat tissues can clarify the contours of the abdominal viscera packed within the abdominal cavity (25). However, the contours of most abdominal viscera, such as the liver, are commonly unclear, limiting the clinical utility of plain abdominal radiography (25). Conversely, lesions and materials generating lower or higher radiodensities can provide good contrast against adjacent abdominal viscera on plain abdominal radiographs. Radiography is superior to the US in identifying metallic foreign bodies causing traumatic reticuloperitonitis, which appear mostly as radiopaque, sharp, linear materials contrasting well with the radiodensities of the thoracoabdominal region, including the reticulum, diaphragm, and heart (17, 48, 53). Full attachment of the magnet with a metallic foreign body and its position partly predicts reticular perforation (53, 111). Despite appearing as a hyperechoic linear structure causing acoustic shadowing on US, US rarely identifies metallic foreign bodies (17, 25). Gas-filled abscesses are detectable as radiolucent structures on abdominal radiographs, providing good contrast to the radiopaque structures of abdominal viscera (53, 111, 112). The formation of a gas-filled or gas-fluid interface mass adjacent to the reticulum is a suggestive radiographic sign of a perforating foreign body (111). Peritoneal effusion makes the contours of abdominal viscera unclear (25). Despite the higher potential of US to distinguish these types of peritoneal effusions, there is no specific radiographic sign to differentiate between transudates, exudates, and modified transudates (25).

Using a positive contrast medium helps distinguish abdominal organs from each other on radiographs of the ruminant abdomen, where the rumen occupies the majority of the space (17). The upper or lower gastrointestinal tracts can be identified radiographically by administering barium sulfate medium via a stomach tube in younger calves and small ruminants (22). However, a disadvantage of this method in ruminants is the time-dependent differences in contrast enhancements in the gastrointestinal tracts; for goats, several minutes to hours are required after oral administration of barium sulfate medium to enhance the forestomach, small intestine and large intestine (22). Additionally, the superimposition of positively enhanced gastrointestinal tracts can result in the images obscuring each other (22). This appears to be why routine abdominal US is replacing gastrointestinal contrast radiography (25). Excretory urography has been previously utilized for small ruminants and young calves, allowing time-dependent, normal contrast enhancement within the renal parenchyma such as the hilus and renal pelvis, followed by contrast medium filling within the lumens of the ureter, and finally, the urinary bladder (112–114). Experimentally induced nephrosis caused the cessation or delay of contrast medium outflow within the urinary tract (113, 114). In a previous case involving a calf with a gas-filled urachal abscess, this technique effectively confirmed no communication between the urinary bladder and the mass (112). Retrograde urethrography and cystography are also available to clarify communication between the urinary bladder and the mass via the persistent urachus (115). These contrast radiographic techniques targeting the urinary tract can complement the limitations of the abdominal US, which sometimes incompletely identifies the urinary tract; the kidney and urinary bladder are partly visible, whereas the ureter is rarely evident.

CT is a valuable imaging modality used for visualizing organs throughout the entire body without superimposition, and it is effective for both for small and large ruminants when available (2, 22, 107, 116). The intravenous injection of contrast medium during CT scanning enhances the ability to distinguish between normal and abnormal structures present within organs (e.g., the liver) by providing differing contrast enhancements between these structures (25, 78, 116). Abdominal CT scanning, combined with gastrointestinal contrast, is particularly useful for evaluating pathological changes within the gastrointestinal lumens (116). For instance, in newborn calves, the umbilical vein can be visualized running between the umbilical cord and the liver along the ventral abdominal walls (117). It has been observed that the umbilical vein typically disappears on CT around 21 days of age, making this an appropriate time frame to detect remnant umbilical veins (117).

The use of CT in 1-month-old Holstein calves presenting with stifle arthritis allowed the identification of purulent omphalophlebitis extending to a liver abscess, aiding in the prognosis. CT revealed pneumothorax as the unilateral accumulation of gas within the left thoracic cavity, effectively identifying infectious lesions in previous CT scans of the thoracic and abdominal regions (44, 78, 112).

In cases of echinococcosis, CT has revealed the intrahepatic formation of various sizes of round hypoattenuating structures, with changes in the thickened and mineralized cystic walls depending on the developmental stages of the disease (25). Using multi-directional reconstructed and three-dimensional (3D) CT images and basic transverse sections, the extent and severity of lesions formed within the organs, such as lung and liver abscesses, can be evaluated. This approach also allows for the assessment of the spatial relationship between lesions and adjacent organs, as well as any mass effects (44, 73, 116).

There are great technical limitations in each of these imaging modalities in clinical practice. In radiography, the limitation-associated factors include the magnitude of X-ray irradiation power generated from the X-ray machine, the sizes of the animals applicable for radiographic examination, and the number of required examiners (44, 107). High X-ray irradiation is required for X-ray transmission through the large body mass of adult cattle and buffaloes (53). The required X-ray conditions are 85–113 kVp and 50–200 mAs, and 90–115 kVp and 50–70 mAs, when taking thoracic and abdominal radiographs for adult large ruminants, respectively (10, 48, 53). In contrast, the exposure conditions are 45–85 kVp and 2–50 mAs for the chest of small ruminants and younger calves (15, 36, 107, 110). Radiographic limitations are also associated with the difficulty in evaluation using two-directional radiographic views (107). Dorsoventral radiographs are very difficult to take for the thoracic and abdominal regions of adult animals with larger dorsoventral depths of these regions (53, 107). Sedation is commonly required to place animals in dorsal recumbency for proper positioning to take dorsoventral radiographs (25).

US has limited ability to demonstrate lesions presenting at depths >10 cm, even when using a transducer with lower US frequency (2, 36). When scanning large-sized structures, the US can provide fragmented images because the scanning window is too small to scan the whole body (2, 25). Therefore, it is required to visualize the full length of targeted structures based on the sequential US obtained by scanning while moving the transducer gently and slowly across the skin's surface. Successful results from this scanning method depend on the skill levels of the sonographer.

CT has limitations regarding acceptable body sizes and weights in examined animals. Adult cattle are not commonly applicable as the examined target for CT scanning due to their large body mass unless using a specific CT machine with a larger CT gantry and scanning table capable of tolerating heavy body weight (117, 118). Deep anesthesia is commonly required for CT scanning, whereas sedation can sufficiently immobilize an animal for CT scanning when using a multidetector CT machine that allows for shortened scan times (25, 109). Additionally, endotracheal intubation is recommended to control breathing during CT scanning, such as breath-hold techniques, because breath motion can generate motion artifacts and reduce image quality (22, 107, 118), despite previous reports suggesting that motion-associated imaging quality is not usually poor for diagnostic assessment (44).

Therefore, routine use of US should be combined with radiography to enhance sensitivity in detecting infectious lesions within the chest and abdomen (17, 22). CT would be the advanced imaging modality to complement the routine use of US and radiography.

3D US is an advanced imaging technique that can provide various directional axes of organs more accurately than two-dimensional US (119). Previous uses of 3D US aimed to measure bovine mammary glands when scanned percutaneously (120) and fetuses within a gravid uterus when scanned transrectally (119). In terms of the clinical applicability of this method for diagnosing infections and thoracic and abdominal diseases, a freehand manner of moving the transducer slowly along targeted structures may be suitable for demonstrating immovable lesions such as abscess formation within the lung and liver and infectious foci in the urinary tracts and umbilicus (120).

## 5 Conclusion

The combined use of US, radiography, and CT can help overcome the limitations of individual imaging modalities in both large and small ruminants. To further enhance the diagnostic efficacy of these imaging techniques, whether used in routine practice or advanced settings, it is crucial to incorporate 3D or four-dimensional US machines and introduce diagnostic assistance systems, such as artificial intelligence algorithms. Additionally, it is important to recognize that extensive basic data from previous ruminant cases is essential for maximizing the potential of these advanced technologies. Therefore, ongoing integrated research is necessary to continually update and refine the range of abnormal imaging findings associated with both common and rare diseases while exploring newly developed imaging techniques, such as advanced US scanning methods.
